# Proximal Landing Zone’s Impact on Outcomes of Branched and Fenestrated Aortic Arch Repair

**DOI:** 10.3390/jcm14103288

**Published:** 2025-05-08

**Authors:** Petroula Nana, Konstantinos Spanos, Giuseppe Panuccio, José I. Torrealba, Fiona Rohlffs, Christian Detter, Yskert von Kodolitsch, Tilo Kölbel

**Affiliations:** German Aortic Center, Department of Vascular Medicine, University Heart and Vascular Center UKE Hamburg, 20251 Hamburg, Germany; spanos.kon@gmail.com (K.S.); giuseppe.panuccio@gmail.com (G.P.); jitorrealba@gmail.com (J.I.T.); fionarohlffs@web.de (F.R.); detter@uke.de (C.D.); kodolitsch@uke.de (Y.v.K.); tilokoelbel@googlemail.com (T.K.)

**Keywords:** aortic arch, endovascular aortic repair, landing zone, mortality, stroke

## Abstract

**Background/Objectives:** The impact of the proximal landing zone has not been investigated in fenestrated and branched endovascular aortic arch repair (f/bTEVAR). This study aimed to analyze the f/bTEVAR outcomes in patients with non-native (nNPAL) vs. native proximal aortic landing (NPAL). **Methods**: The STROBE statement was followed in order to conduct a single-center retrospective analysis of patients with nNPAL vs. NPAL managed, from 1 September 2011 to 30 June 2022, with f/bTEVAR. The primary outcomes were technical success, 30-day mortality and stroke. **Results**: A total of 83 patients with nNPAL vs. 126 patients with NPAL were included. Among the nNPAL group, 34 (39.7%) underwent previous aortic arch replacement and the remaining underwent an ascending aortic replacement. The nNPAL patients were more commonly treated for chronic dissections (nNPAL: 70.6% vs. NPAL: 21.6%, *p* < 0.001), presented a more proximal disease (zone 0: nNPAL: 27.7% vs. NPAL: 7.1%, *p* < 0.001; zone 1: nNPAL: 50.6% vs. NPAL: 10.2%, *p* < 0.001) and received more triple-branch devices (nNPAL: 16.9% vs. NPAL: 3.2%, *p* < 0.001), with a higher rate of Ishimaru zone 0 landing (nNPAL: 86.8% vs. NPAL: 51.6%, *p* < 0.001). Technical success (nNPAL: 98.8% vs. NPAL: 94.4%, *p* = 0.07) and 30-day mortality (nNPAL: 6.0%, vs. NPAL: 11.9%, *p* = 0.16) were similar. Stroke was lower among nNPAL patients (nNPAL: 4.8% vs. NPAL: 13.5%, *p* = 0.04). A multivariate regression analysis confirmed nNPAL as an independent protector for stroke (*p* = 0.002). Survival (log rank: *p* = 0.02) was higher within the nNPAL group at 24 months. **Conclusions**: f/bTEVAR in patients with nNPAL zone showed encouraging outcomes. Despite more proximal landing in zone 0, stroke was significantly lower when compared to NPAL patients.

## 1. Introduction

Endovascular repair with fenestrated and branched devices has shown promising outcomes in terms of early mortality in high-risk patients with diseases affecting the aortic arch [1,2,3,4]. Despite the associated decreased early morbidity, compared to open arch repair (OAR), the related stroke rate remains alarming and seems to affect patients’ survival [1,2,3,4,5,6]. Various measures, such as device CO_2_ flushing, have provided encouraging findings in previous studies and have become the standard of care [7,8]. However, the further modifications of the techniques and evolution of new devices, as well as different access approaches, may be needed to ameliorate the fenestrated and branch aortic arch endovascular repair (f/bTEVAR) outcomes and decrease the related cerebrovascular morbidity [9,10].

According to the latest recommendations, total endovascular management in patients with adequate anatomy may be justified [11]. The proximity of landing to the ascending aorta has shown to affect stroke rates, with landing at the Ishimaru zone 0 being related to higher cerebrovascular morbidity. Initial experiences with endovascular aortic arch repair reported a stroke rate of up to 15% and a major stroke of up to 5% [5,12]. The impact of the proximal landing zone‘s nature—native vs. previously repaired—has not been investigated, while previous data on patients with replaced aortas after type A aortic dissections showed low stroke rates, raising the question of proximal landing zone impact on f/bTEVAR outcomes [13,14,15].

The aim of the current study is to investigate the comparative outcomes of f/bTEVAR in patients with a previously replaced proximal landing zone (non-native proximal landing zone; nNPAL) vs. patients with a native proximal landing zone (NPAL) at 30 days and during the available follow-up.

## 2. Materials and Methods

### 2.1. Study Design

A single-center retrospective analysis of patients managed from 1 September 2011 to 30 June 2022 who had custom-made fenestrated and branched endografts for lesions affecting the aortic arch and landing within nNPAL vs. NPAL zones was conducted. Patients with nNPAL were compared to the NPAL cohort, which has been previously analytically described [16]. The data of the NPAL cohort were re-analyzed via comparisons to the data of patients managed with f/bTEVAR and nNPAL. The STrengthening the Reporting of OBservational studies in Epidemiology (STROBE) statement was followed [17].

### 2.2. Patient Population

Elective und urgent cases with nNPAL vs. NPAL, managed with f/bTEVAR for pathologies involving the aortic arch, were included. All cases were evaluated in a multidisciplinary aortic board, including cardiovascular experts and anesthesiologists, to assist decision making [4]. Patients with genetic aortic syndromes (GAS) were included, as before any endovascular management, a proximal aortic repair was performed [18,19]. Patients treated with physician-modified endografts, including in situ-modified devices or the parallel graft technique, were omitted.

Custom-made f/bTEVAR devices (CMDs) based on the Zenith platform (Cook Medical, Bloomington, IN, USA) were used, following the manufacturer’s instructions on proximal landing [4]. A proximal diameter ≤38 mm and a landing zone of at least 30 mm were used for patients needing repair for a degenerative disease while a landing zone length of 20–25 mm was considered adequate in patients managed for aortic dissections [4]. An oversizing of 20% was applied in all cases, except for patients with aortic dissection where an oversizing up to 10% was used. Patients treated under the urgent setting were managed with CMDs, which were readily available as stock. These devices were either designed for this specific patient as part of a scheduled elective procedure, which had to performed earlier due to patient’s symptoms or aortic rupture, or were designed for another patient whose anatomy conformed in terms of length of coverage, diameter of proximal landing zone and target vessel clock position to the anatomy of the patient needing the urgent repair [20,21]. Details on endograft configuration, device selection and technique have been published previously [4]. Fenestrated devices were mainly used for distal arch lesions affecting the inner curvature and landing targeting the transverse arch, while branched devices were preferably used for pathologies affecting the outer curvature and more proximal landing in Ishimaru zone 0 [4].

For the introduction of the main device, transfemoral access was used, while for the intended target vessels (TVs), either a transfemoral or an upper access was performed [4]. For the TVs, anterograde access was used in either the case of a retrograde branch device or in triple branch devices for the revascularization of the left common carotid artery (LCCA), using steerable sheaths. Transapical access was used as a bailout when transfemoral or upper access did not permit the advancement of the main device or innominate artery’s (IA) bridging limb. Cervical debranching was performed according to the patient’s anatomy and planned repair [22]. No debranching was performed using a sternotomy. When a left carotid-subclavian (LSA) bypass was used for debranching, the bridging stent intended for the LCCA was delivered using a left trans-brachial access [4]. Bridging stent selection for the LCCA and LSA was at the discretion of the operator, with balloon-expandable, self-expanding or a combination of both covered stents being applied [4]. IAs were mainly stented with custom-made bridging limbs (Cook Medical, Bloomington, IN, USA). Relining with bare metal stents was decided intra-operatively.

### 2.3. Data Collection

A dedicated local database was created for the collection of pre-, intra-, and post-operative patient information. Computed tomography angiography (CTA) was performed before discharge, at 12 months, and yearly thereafter, while the surveillance protocol differed depending on the imaging findings. A 3-month re-evaluation was performed especially for patients with a type Ia endoleak detected in the pre-discharge CTA. Small endoleaks with no sac expansion may have been treated with a “watch and wait” approach after patients were provided with information about the potential risk and benefits. Larger endoleaks with sac expansion or endoleaks related to false lumen retrograde flow were managed using coil embolization or false lumen occlusion using dedicated endografts. Survival and freedom from endoleak, TV stenosis or occlusion and re-intervention during follow-up were recorded. All data were pseudonymized and introduced in the same database. General hospital records were periodically assessed to identify potential adverse events. This study complied with the Declaration of Helsinki and no further approval was required from the local ethics committee due to its retrospective design and unidentifiable information in accordance with current state law.

### 2.4. Definitions

The location of the largest diameter of any lesion or the diameter setting the indication for repair was used to identify the lesions’ location within the different aortic segments. In particular, if the diameter of an aneurysm was over 55 mm within the aortic arch, this was primarily classified as an arch aneurysm, irrespective of its extension. When the widest portion of the aneurysm was in the descending thoracic or thoracoabdominal aorta, the case was registered as a thoracic (TAA) or thoracoabdominal (TAAA) aortic aneurysm, respectively. The classification of the aortic arch type was performed according to Madhwal, et al. [23]. The landing zone was defined according to Ishimaru at the location of the most proximal end of the endograft [24].

Technical success was defined as a composite of successful access to the arterial system, the delivery and deployment of the main endograft and modular components to the intended site, side branch catheterization and bridging stent deployment with patent intended TVs and the absence of type I or III endoleaks at the completion of angiography that extends beyond 30 days [25]. Post-operative adverse events, including strokes, spinal cord ischemia (SCI) and acute kidney injury (AKI), were defined and classified according to the Society of Vascular Surgery reporting standards [25]. Early experience was identified as the time period from 2011 to 2015, which was used in order to investigate whether there was any potential impact on the findings of the current analysis.

### 2.5. Outcomes

The primary outcomes were the comparative technical success, mortality and stroke in patients with nNPAL vs. patients with NPAL at 30 days. Follow-up outcomes were also analyzed.

### 2.6. Statistical Analysis

Normally distributed continuous data were reported as mean ± standard deviation and non-normally distributed were reported as median values with range and IQR. Categorical data were expressed as absolute numbers and percentages. A chi-square test was used for categorical data comparison. Independent two-sample *t* tests were used for normally distributed continuous variables, and the Mann–Whitney U test was used for non-normally distributed continuous and ordinal variables. A univariate regression analysis was performed to investigate potential factors [early experience (2011–2015), sex, urgency, ruptures, diabetes, combined previous stroke/transient ischemic attack (TIA), proximal landing zone nature, landing to Ishimaru zones 0 and 1, endograft configuration, post-operative stroke, retrograde type A aortic dissection, pericardial effusion, acute kidney injury (AKI) and respiratory failure] related to 30-day mortality and stroke. Only the factors detected as significant within the univariate analysis were introduced in the multivariate regression analysis. The *p* value was considered significant when it was <0.05. No correction for multiple hypothesis testing was applied. The sample size was able to vary based on the analysis and no imputation of missing data was performed, since both the missing categorical and continuous variables were infrequent. Kaplan–Meier estimates, and log rank was used to assess the follow-up outcomes. Statistical analysis was performed using SPSS 29.0 for Windows software (IBM Corp, Armonk, NY, USA).

## 3. Results

### 3.1. Patient Cohort

A total of 83 patients with nNPAL underwent f/bTEVAR (total cohort 209 patients; 126 patients with NPAL) within the study period [16]. Among the nNPAL cohort, 41 (49.4%) were males with a mean age 68.3 ± 5.5 years. Seven (8.4%) patients were managed urgently; three patients had ruptures (3.6%). For the NPAL group, 88 (69.8%) patients were males, and the mean age was 70.8 ± 4.2 years. Twenty-three (18.3%) patients were managed urgently. The comparative analysis on baseline characteristics between nNPAL vs. NPAL patients is presented in Table 1. Only male sex (nNPAL: 49.4% vs. NPAL: 69.8%, *p* = 0.003) and diabetes (nNPAL: 6.0% vs. NPAL: 16.7%, *p* = 0.02) were less common within the nNPAL group. The patients’ baseline characteristics are presented in Table 1.

Regarding previous aortic interventions (Table 2), patients with nNPAL had a greater number of previous aortic valve repairs (nNPAL: 36.1% vs. NPAL: 1.6%, *p* < 0.001), including mechanical aortic valves (nNPAL: 14.4% vs. NPAL: 0.8%, *p* = 0.02). Comparative patient lesion distribution is presented in Table 2. Regarding proximal disease extension according to Ishimaru zones, 27.7% of the nNPAL group presented zone 0 extension vs. 7.1% in the NPAL group and 50.5% in zone 1 for the nNPAL group vs. 10.2% for the NPAL group (*p* < 0.001). Arch type I was more common with the nNPAL group (54.2% vs. 31.0%, *p* < 0.001) while type III was more common within the NPAL group (4.8% vs. 14.2%, *p* = 0.03).

### 3.2. Previous Aortic History and Current Disease

For the nNPAL group, 34 (39.7%) patients had undergone open aortic arch repair while the remaining patients had previously undergone ascending aortic replacement. Thirty patients (36.1%) had undergone previous aortic valve repair; among them, twelve had a mechanical aortic valve. Patients with nNPAL presented higher rates of aortic valve repair (nNPAL: 36.1% vs. NPAL: 1.6%, *p* < 0.001), including mechanical aortic valves (nNPAL: 14.4% vs. NPAL: 0.8%, *p* = 0.02). No difference was detected between groups when analyzed for more distal previous aortic repair, as presented in Table 2.

Patients with nNPAL were more commonly treated for aortic dissections (nNPAL: 63.9% vs. NPAL: 25.4%, *p* < 0.001), including post-dissection arch aneurysms (nNPAL: 70.6% vs. NPAL: 21.6%, *p* < 0.001, Table 2). Subsequently, nNPAL patients presented more proximal aortic disease (zone 0: nNPAL: 27.7% vs. NPAL: 7.1%, *p* < 0.001; zone 1: nNPAL: 50.6% vs. NPAL: 10.2%, *p* < 0.001), with 78.3% (vs. NPAL: 17.3%) being managed for aortic diseases extending to zone 0 and 1 and receiving more triple-branched devices (nNPAL: 16.9% vs. NPAL: 3.2%, *p* < 0.001).

### 3.3. Device Configuration

A total of 11 (13.3%) patients were managed with fTEVAR and 72 (86.7%) were managed with bTEVAR in the nNPAL group vs. 72 (57.1%) fTEVAR and 44 (34.9%) with bTEVAR in the NPAL group, as presented in Table 3. BTEVAR was more commonly applied in patients with nNPAL (*p* < 0.001) and fTEVAR was more commonly applied in the NPAL group (*p* < 0.001). Among the bTEVAR devices, for the nNPAL group, 14 (19.4%) received a triple-branch device, while in the NPAL group (*p* < 0.001), only 4 (9.0%) received a triple-branch device. All planned branches and fenestrations were connected to their planned TV. Debranching and distal extension procedures were more common with the nNPAL group (*p* < 0.001, both), as is presented in Table 3. False lumen endografts (FLE) were similarly applied (*p* = 0.14). Patients with nNPAL presented a higher rate of proximal landing in Ishimaru zone 0 (zone 0: nNPAL: 86.8% vs. NPAL: 51.6%, *p* < 0.001) vs. patients with NPAL that presented a higher rate in landing in zone 1 (nNPAL 10.8% vs. NPAL: 46.0%, *p* < 0.001). No difference was detected for zone 2 (*p* = 0.98).

### 3.4. Thirty-Day Outcomes

Technical success (nNPAL: 98.8% vs. NPAL: 94.4%, *p* = 0.07) and 30-day mortality (nNPAL: 6.0%, vs. NPAL: 11.9%, *p* = 0.16) were similar between groups. The univariate analysis identified pre-operative stroke and TIA (*p* = 0.001), ruptures (*p* = 0.02), technical failures (*p* < 0.001), post-operative strokes (*p* < 0.001; major stroke, *p* < 0.001), pericardial effusion (*p* < 0.001), AKI (*p* < 0.001) and respiratory failure (*p* < 0.001) as related to 30-day mortality. The multivariate regression analysis confirmed the post-operative major strokes (*p* < 0.001), pericardial effusions (*p* < 0.001) and respiratory failures (*p* = 0.043) as independently related. Early experience was not a predictor for mortality (*p* = 0.74).

The comparative post-operative outcomes showed that stroke (nNPAL: 4.8% vs. NPAL: 13.5%, *p* = 0.04) was lower among patients with nNPAL. The difference in major stroke rate was not statistically significant (nNPAL: 2.4% vs. NPAL: 7.9%, *p* = 0.09, Table 4). A univariate analysis on stroke outcomes identified previous cerebrovascular events (stroke and TIA; *p* = 0.02) and landing in zone 0 (*p* = 0.01) and 1 (*p* = 0.02) as related to stroke, while nNPAL (*p* = 0.04) and TBAD (*p* = 0.02) were negatively related. The multivariate regression analysis confirmed nNPAL as an independent protective factor for stroke (*p* = 0.002) and previous cerebrovascular events as an independent predictor for stroke (*p* = 0.03). Early experience was not related to stroke (*p* = 0.88).

Patients with nNPAL were more prone to present acute kidney injury post-operatively (nNPAL: 14.4% vs. NPAL: 5.5%, *p* = 0.03). A significant difference was detected in terms of type Ia and Ib endoleaks, with higher rates among the nNPAL group (nNPAL: 16.9% vs. NPAL: 6.3%, *p* < 0.001 and nNPAL: 12.0% vs. NPAL: 3.9%, *p* < 0.001, respectively). The post-operative adverse events are presented in Table 4.

All TVs were patent on pre-discharge CTAs for both groups. Endoleak distribution in the predischarge CTA is presented in Table 4. Type I endoleaks were more common with the nNPAL group (nNPAL: 32.5% vs. NPAL: 11.1%, *p* < 0.001), including type Ia: nNPAL: 14 (16.9%) vs. NPAL: 8 (6.3%), *p* = 0.02 and type Ib: nNPAL: 10 (12.0%) vs. NPAL: 5 (3.9%), *p* = 0.03. None of them required a secondary intervention. After 30 days, two patients, presented with persisting (after 30-day follow-up) type Ia endoleak, one in each group. Twenty-five patients (30.1%) in the nNPAL group underwent a secondary reintervention within 30 days vs. 24 (10.0%) in the NPAL group (*p* = 0.06). Most reinterventions were access related for both groups:—nNPAL: 19 (22.9%) vs. NPAL: 28 (22.2%), *p* = 0.90.

### 3.5. Follow-Up Outcomes

**Figure 1:**
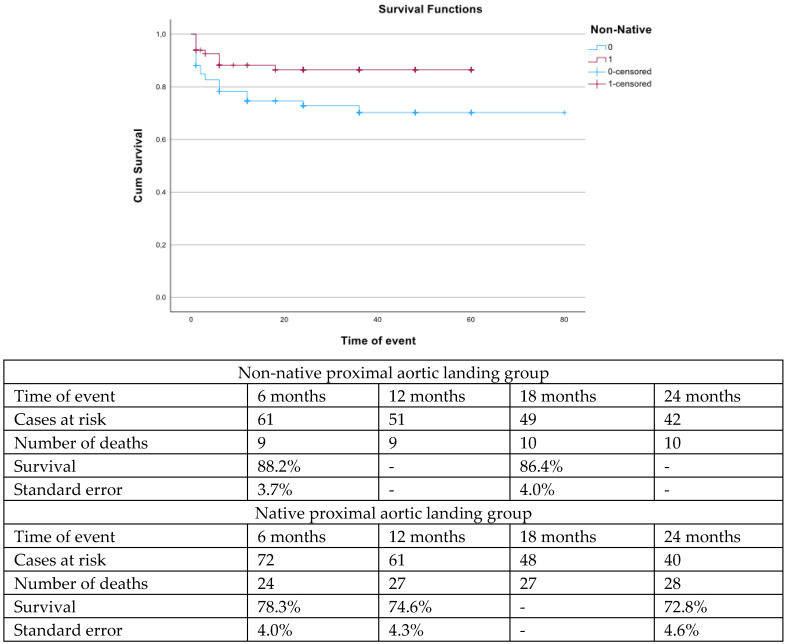
The estimated survival was higher among patients with non-native proximal landing zone vs. patients with native proximal landing zone (log rank, *p* = 0.02) managed with fenestrated and branched endovascular aortic arch repair.

**Figure 2:**
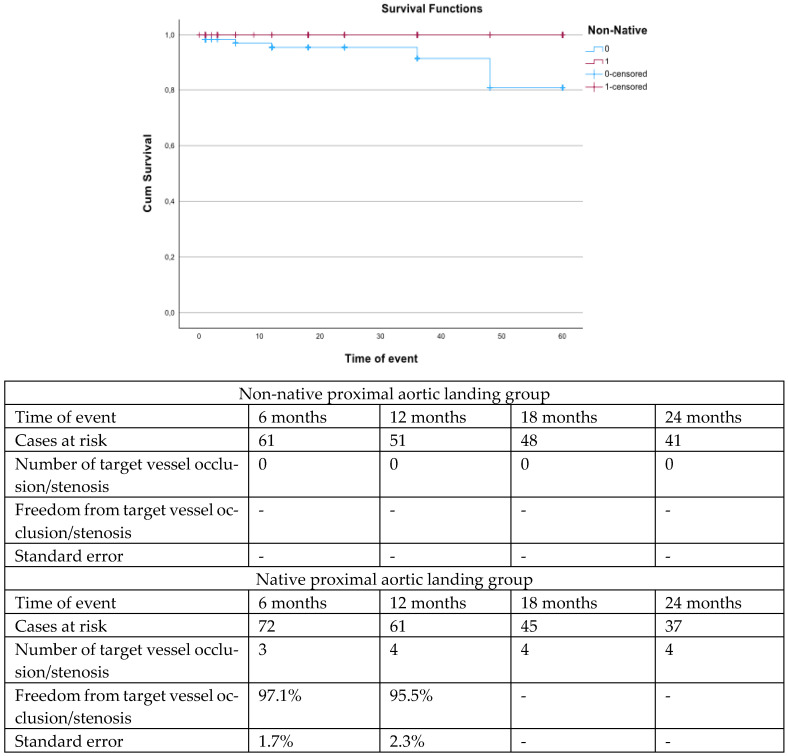
The estimated freedom from target vessel occlusion/stenosis was higher among patients with non-native proximal landing zone vs. patients with native proximal landing zone (log rank, *p* = 0.01).

The mean follow-up was 27.0 ± 14.3 months. During follow-up, patients with nNPAL presented better survival rates (log rank; *p* = 0.02, Figure 1) and higher freedom from TV occlusion or stenosis (log rank: *p* = 0.01, Figure 2).

**Figure 3:**
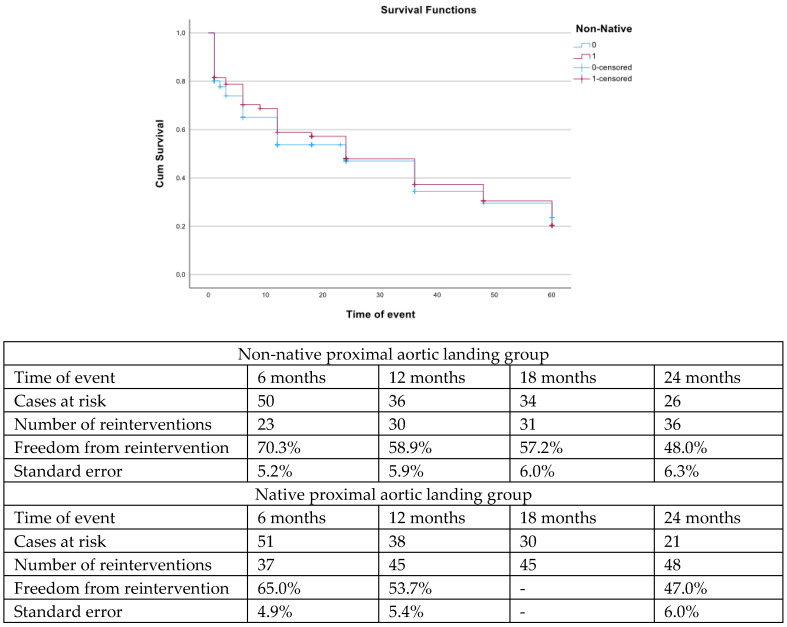
The estimated freedom from reintervention was similar between patients with non-native proximal landing zone vs. patients with native proximal landing zone (log rank, *p* = 0.75).

**Figure 4:**
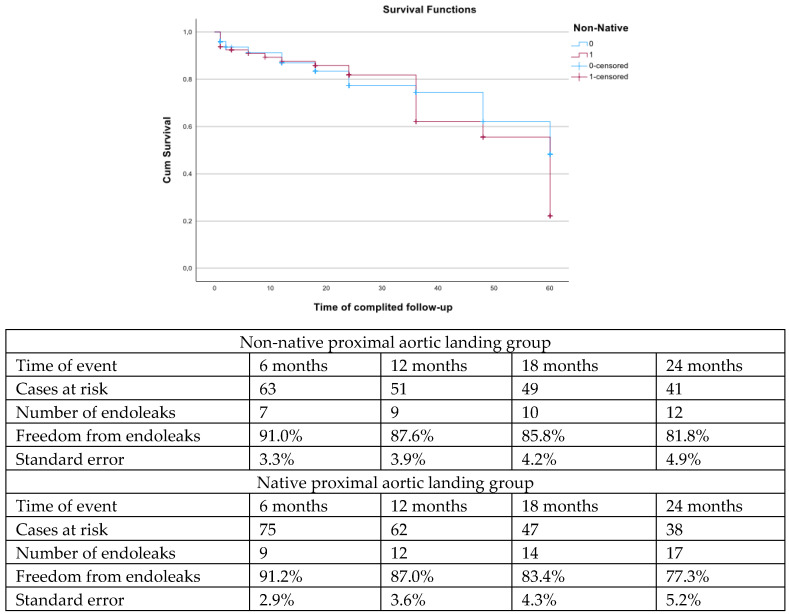
The estimated freedom from endoleak was similar between patients with non-native proximal landing zone vs. patients with native proximal landing zone (log rank, *p* = 0.34).

The estimated freedom from reintervention was 57.2% (SE 6.0%) for the nNPAL vs. 53.7% (SE 5.4%) for the NPAL group at 18 months of follow-up (log rank; *p* = 0.75, Figure 3). Freedom from endoleak was also similar between groups (log rank; *p* = 0.34, Figure 4).

## 4. Discussion

Endovascular aortic arch repair has been applied in high-risk patients with encouraging early outcomes [2,3,4,9,26,27]. However, stroke remains an issue, with occurrence rates of up to 10% [26,27,28]. This analysis showed that when applied among patients with previously replaced aortic segments, early outcomes are favorable, with an overall stroke rate of 4.8% and a major stroke rate less than 2.5%, respectively. These findings are supported by previous publications focusing on patients treated endovascularly after open repair for type A aortic dissections and represent a similar cohort [13]. The follow-up outcomes signify that regardless of the favorable early outcomes, continuous surveillance is of major importance, as reintervention affected almost half of patients during the mid-term follow-up [1].

The latest guidelines for aortic disease from the American Heart Association and American College of Cardiology suggest endovascular aortic arch treatment in high-risk patients with adequate anatomy [11]. However, these recommendations are not stratified regarding the impact of proximal landing zone nature and patients that underwent previous OAR, potentially due to a lack of studies in the literature at that moment. This analysis showed that when landing to zone 0 is performed in a non-native segment, the outcomes are favorable when compared to more distal native landing zones, especially in terms of stroke. On the other hand, open aortic arch repair after previous ascending aortic replacement is accompanied by mortality and stroke rates of around 14% according to single-center limited data, making less invasive endovascular options very attractive [29].

Recent meta-analytic data of patients managed with endovascular—fenestrated or branched—repair for various aortic arch pathologies confirmed an over 95% technical success rate [30]. The current cohort showed that landing zone’s nature potentially does not affect early technical success, with similar rates, of over 94%, in both groups. Despite the fact that almost 75% of patients with repaired type A aortic dissection have been shown to be eligible for endovascular arch repair, it should be mentioned that type Ia endoleaks were higher in the predischarge CTA among nNPAL patients in the current cohort; this is potentially driven by the higher rate of Ishimaru zone 0 landing. The mismatch of length between the outer and inner aortic curve and the angle created in the inner curvature may affect the apposition of the endograft, creating a bird beak effect in patients with landing in zone 0. In addition, the less optimal apposition of a second endograft onto the stiff surface of a previous, potentially infolded, Dacron graft cannot be excluded as a factor of compliance mismatch leading to higher endoleak Ia rates [9,31]. However, more than two thirds of these endoleaks disappeared within the first month after f/bTEVAR, showing that most of them are self-limited within the early follow-up, without needing any secondary intervention.

F/bTEVAR gained popularity due to the decreased early mortality in patients considered as high-risk for conventional OAR [27,30]. In this analysis, the mortality rate among nNPAL patients was at 6.0%; this was lower than the NPAL cohort, without achieving statistical significance. When taking into consideration that redo aortic arch operations may be far more technically complex, the findings of using an endovascular approach in these patients may be highly encouraging [32,33]. The limited published experience has detected redo sternotomy in patients with previous proximal aortic repair as a factor related to early and long-term mortality [34]. Similarly, minimal invasive approaches for valve replacement in patients having a history of sternotomy showed significant benefit in terms mortality and AKI, as well as length of hospital stay [35].

When comparing patients with nNPAL vs. NPAL, a significant difference was identified in terms of stroke; the major stroke rate also presents lower rates among nNPAL cases but does not achieve a statistical significance. Stroke is a significant complication, affecting mortality in patients undergoing endovascular aortic arch repair [28,36]. Stroke rates in mixed cohorts have been reported as ranging between 0 and 42.9%, and are affected by the atherosclerotic burden, air released from the endovascular device and a variety of factors leading to cerebral perfusion insufficiency [36]. The presence of a previous graft, as well as the fact that the vast majority of nNPAL patients were managed for aortic dissections, and not degenerative disease, potentially highlights the role of these two factors and signifies the presence of a cohort at lower risk for stroke. Previous data from aortic arch cases managed endovascularly after initial aortic replacement showed a composite mortality–stroke rate at 4% [13,14].

In addition to lower stroke rates, the absence of retrograde TAAD events within the nNPAL cohort should be acknowledged. This rare complication, which may be fatal in 40% of cases, is completely avoided in patients with previously replaced aortic landing zones [37]. While in previous standard TEVAR analyses, the estimated retrograde TAAD rate is 2.5%, in the NPAL group of the current study, retrograde TAAD affected 3.9% of patients [37,38]. The proximity of repair, within landing in Ishimaru zones 0 and 1, justifies the higher rate of retrograde TAAA compared to standard TEVAR [37].

Despite the acceptable early results, the estimated survival was limited to almost 85% at 18 months for the nNPAL cohort and 75% for the NPAL, showing that despite the minimally invasive nature of the procedure, the targeted population remains at risk for death after repair. This fact could potentially be explained by the high-risk pre-operative profile of the cohort, with more than 83% of cases presenting an ASA score ≥ 3, as shown also in previous studies [39]. However, even in the “fit” open cohorts, the estimated mid-term survival is 85%, showing that both assessments—open and endovascular—target an a priori fragile population [40,41]. The survival among nNPAL patients was higher, potentially affected by the higher rate of urgent cases within the NPAL cohort [42]. Simultaneously, reinterventions affect almost one in two cases within the initial 24 months. While reinterventions at 30 days are related to access adverse events, with a rate of 22% in both groups, reinterventions during follow-up are related to the main aortic repair, including distal extensions. The high rate of patients with underlying thoracoabdominal aneurysms within the cohort in addition to aortic dissection, especially type A, and the need for extensive aortic coverage, can explain the high need for reinterventions during follow-up [13,43]. The findings presented in the current analysis represent the experience of almost a decade of a high volume aortic center and should be interpreted under this scope. FbTEVAR is a technically demanding procedure performed in a high-risk population and significant experience of complex aortic procedures of more distal aortic segments is mandatory for the safe performance of these types of repairs.

### Limitations

Both the sample size and retrospective design of the analysis represent important limitations and should be taken into consideration when interpreting its findings. Potential selection bias cannot be excluded, especially as patients with nNPAL were more commonly treated under elective setting, leading to worse outcomes in the NPAL group. A variety of aortic arch lesions were managed, with a case-by-case approach according to the patient’s specific needs and anatomic characteristics. Other anatomic factors, including the presence of shaggy aorta, aneurysm volume and the presence of an in sac thrombus, arch calcification severity and TV anatomy were not analyzed, but these represent an interesting field needing further analysis. In addition, further information on the previous open aortic procedures in terms of graft type, time of initial repair and anastomosis details were not available and an analysis on their potential impact on fbTEVAR outcomes could not be investigated at this time point. The inclusion of urgent cases potentially led to worse 30-day outcomes but permits a holistic view of endovascular aortic arch repair data. The lack of advanced imaging in stroke diagnosis may have led to an underestimation of the cerebrovascular event rate within the current cohort. Studies using more dedicated imaging modalities, such as diffusion-weighted magnetic resonance imaging and transcranial Doppler may shed more light on the potential mechanism of stroke evolution during fbTEVAR. Despite the fact that the impact of early experience on the main outcomes was investigated, the lack of a specific identification of the learning curve, in addition to the multiple modifications of the devices through time, should be acknowledged. Type II errors should also be acknowledged.

## 5. Conclusions

Endovascular aortic arch repair in patients with a non-native proximal landing zone showed favorable early outcomes, with a major stroke rate at 2.0%; this was significantly lower when compared to patients with native proximal landing. Despite more proximal landing in zone 0, patients with previously replaced landing zones seem to represent a lower risk cohort for early adverse events when managed with f/bTEVAR. Future multicenter studies of a prospective nature are of major importance to clarify fbTEVAR outcomes and the potential factors that could affect them, such as landing zones.

## Figures and Tables

**Table 1:** Comparative baseline characteristics of patients managed with fenestrated and branched endovascular aortic arch repair with non-native (nNPAL) vs. native (NPAL) landing zones. Footnotes: ASA: American Society of Anesthesiologists; eGFR: estimated glomerular function rate.

Variables (N, %, Mean ± SD, Median; IQR)	nNPAL Group (83 Patients)	NPAL Group (126 Patients)	*p*
Age (years)	68.3 ± 5.5	70.8 ± 4.2	0.08
Males	41 (49.4)	88 (69.8)	0.003
Tobacco use	31 (37.3)	55 (43.7)	0.37
- Active tobacco use	13 (15.7)	32 (25.4)	0.09
Hypertension	75 (90.3)	116 (92.1)	0.68
Diabetes mellitus	5 (6.0)	21 (16.7)	0.02
Dyslipidemia	38 (45.8)	66 (53.2)	0.35
Coronary artery disease	20 (24.1)	41 (32.5)	0.19
Chronic heart failure	8 (9.6)	12 (9.5)	0.97
Chronic obstructive pulmonary disease	15 (18.1)	25 (19.8)	0.75
Chronic kidney disease	7 (8.4)	17 (13.5)	0.26
- Creatinine (mg/dL)	1.1 ± 0.2	1.2 ± 0.3	0.06
- eGFR (mL/min)	72.0 ± 11.0	67.2 ± 9.7	0.13
- Dialysis	0 (0.0)	2 (1.6)	0.36
Stroke	10 (12.0)	10 (7.9)	0.32
Transient ischemic attack	7 (8.4)	6 (4.8)	0.26
Peripheral arterial disease	5 (6.0)	18 (14.3)	0.06
ASA score	3 (IQR 0)	3 (IQR 0)	0.68
ASA score ≥ III	71 (85.5)	104 (82.5)	0.56
Urgent setting	7 (8.4)	23 (18.3)	0.04
- Ruptured aneurysm	3 (3.6)	12 (9.5)	0.11
- Symptomatic	4 (4.8)	11 (8.7)	0.28

**Table 2:** Aortic lesion classification and proximal extension of the disease following Ishimaru zones in patients with non-native (nNPAL) vs. native proximal landing zone (NPAL) treated with fenestrated and branched endovascular aortic arch repair. Footnotes: EVAR: endovascular abdominal aortic repair; f/bEVAR: fenestrated and branched endovascular aortic repair; f/bTEVAR: fenestrated and branched thoracic endovascular aortic repair; TAAA: thoracoabdominal aortic aneurysm; TAAD: type A aortic dissection; TBAD: type B aortic dissection; TEVAR: thoracic endovascular aortic repair.

Variable (N, %)	nNPAL Group (83 Patients)	NPAL Group (126 Patients)	*p*
Previous aortic repair			
Aortic valve repair	30 (36.1)	2 (1.6)	<0.001
- Mechanical aortic valve	12 (14.4)	1 (0.8)	<0.001
f/bTEVAR	0 (0.0)	1 (0.8)	0.54
TEVAR	6 (7.2)	19 (15.1)	0.09
Open thoracic aortic repair	2 (2.4)	4 (3.2)	0.75
f/bEVAR	1 (1.2)	6 (4.7)	0.16
EVAR	6 (7.2)	5 (3.9)	0.30
Open abdominal aortic repair	6 (7.2)	13 (10.2)	0.45
Aortic lesion distribution			
Ascending aneurysms	3 (3.6)	1 (0.8)	0.15
Arch aneurysms	51 (61.4)	37 (29.4)	<0.001
- Degenerative	15 (29.4)	29 (78.4)	0.39
- Dissection	36 (70.6)	8 (21.6)	<0.001
Penetrating aortic ulcer	1 (1.2)	33 (26.2)	<0.001
- Related to aortic dissection	0 (0.0)	1 (3.0)	0.54
Intramural hematoma	2 (2.4)	1 (0.8)	0.37
- Related to aortic dissection	0 (0.0)	1 (100.0)	0.54
Pseudoaneurysms	5 (6.0)	11 (8.7)	0.47
- Related to aortic dissection	1 (20.0)	2 (18.2)	0.82
TAAA type I and II	19 (22.9)	39 (31.0)	0.20
- Degenerative	8 (42.1)	21 (53.8)	0.15
- Dissections	11 (57.9)	18 (46.2)	0.83
Thoracic aortic aneurysms	2 (2.4)	4 (3.2)	.
- Degenerative	1 (50.0)	2 (50.0)	0.82
- Dissection	1 (50.0)	2 (50.0)	0.82
Any aortic dissection	53 (63.9)	32 (25.4)	<0.001
Chronic aortic dissection	49 (59.0)	30 (23.8)	<0.001
- Chronic TAAD	1 (2.0)	4 (3.2)	0.36
- Chronic TBAD	48 (98.0)	26 (20.6)	<0.001
Acute aortic dissection	4 (4.8)	2 (1.6)	0.17
- Acute TAAD	0 (0.0)	0 (0.0)	-
- Acute TBAD	4 (100.0)	2 (100.0)	0.17
Proximal extension of the disease			
Zone 0	23 (27.7)	9 (7.1)	<0.001
Zone 1	42 (50.6)	13 (10.3)	<0.001
- Zone 0 and 1	65 (78.3)	22 (17.3)	<0.001
Zone 2	12	72 (57.1)	<0.001
Zone 3	6	32 (25.4)	<0.001
Arch type			
Type I	45 (54.2)	39 (31.0)	<0.001
Type II	34 (41.0)	69 (54.8)	0.06
Type III	4 (4.8)	18 (14.2)	0.03

**Table 3:** Device configuration among patients treated with fenestrated and branched endovascular aortic arch repair in nNPAL vs. NPAL zone. Footnotes: bTEVAR: branched thoracic endovascular aortic repair; FLE: false lumen endograft; fTEVAR: fenestrated thoracic endovascular aortic repair: LCCA: left common carotid artery; LSA: left subclavian artery; RCCA: right common carotid artery; RSA; right subclavian artery.

Variable (N, %)	nNPAL (83 Patients)	NPAL (126 Patients)	*p*
fTEVAR	11 (13.3)	72 (57.1)	<0.001
Only fenestrations	6 (7.2)	10 (13.9)	0.85
Only scallop	0 (0.0)	5 (6.9)	0.16
Fenestration and scallop	5 (6.0)	57 (79.2)	<0.001
bTEVAR	72 (86.7)	44 (34.9)	<0.001
- Triple branch devices	14 (16.9)	4 (9.0)	<0.001
- Double branch devices	52 (62.7)	37 (29.7)	<0.001
- Single branch devices	6 (7.2)	3 (2.4)	0.09
-LSA branch device	0 (0.0)	10 (13.9)	0.03
Adjacent procedures			
Debranching of supra-aortic trunks	65 (78.3)	68 (54.0)	<0.001
- LCCA-LSA bypass	57 (68.7) *	60 (47.6) **	-
- LSA transposition	2 (2.4)	6 (4.8)	-
- RCCA-RSA	2 (2.4)	2 (1.6)	-
- Axillo-axillary bypass	4 (4.8)	1 (0.8)	-
Ascending thoracic component	0 (0.0)	7 (5.5)	0.08
Distal thoracic extension	65 (78.3)	68 (54.0)	<0.001
FLE (among patients managed for aortic dissection)	27/53 (50.9)	11/32 (34.4)	0.14

* In seven cases, a concomitant RCCA-RSA bypass was performed, while in one case, a left axillo-axillary bypass was performed; ** In four cases, a concomitant RCCA-RSA bypass was performed.

**Table 4:** Comparative 30-day post-operative outcomes between patients with non-native (nNPAL) vs. native (NPAL) proximal aortic landing zones. Footnotes: CTA: computed tomography angiography; UEA: upper extremity access.

Variable (N, %)	nNPAL Group (83 Patients)	NPAL Group (126 Patients)	*p*
Technical success	82 (98.8)	119 (94.4)	0.07
Mortality	5 (6.0)	15 (11.9)	0.16
Stroke	4 (4.8)	17 (13.5)	0.04
- Major stroke	2 (2.4)	10 (7.9)	0.09
- Minor stroke	2 (2.4)	7 (5.5)	0.27
Spinal cord ischemia	3 (3.6)	7 (5.5)	0.52
- Grade 1	0 (0.0)	3 (2.4)	0.36
- Grade 2	3 (3.6)	3 (2.4)	0.60
- Grade 3	0 (0.0)	1 (0.8)	0.77
- Complete recovery	1 (1.2)	4 (3.2)	0.36
- Late evolution	0 (0.0)	1 (0.8)	0.77
- Cerebrospinal fluid drainage	9 (10.4)	28 (22.2)	0.03
Retrograde type A dissection	NA	5 (3.9)	-
Congestive heart failure	2 (2.4)	5 (3.9)	0.54
Pericardial effusion	2 (2.4)	7 (5.5)	0.27
Acute kidney injury	12 (14.4)	7 (5.5)	0.03
- Permanent Dialysis	1 (1.2)	0 (0.0)	0.76
Reinterventions	25 (30.1)	24 (19.0)	0.06
Access complications	19 (22.9)	28 (22.2)	0.90
- UEA complications	6 (7.2)	11 (8.7)	0.70
- Access-related reinterventions	14 (16.9)	16 (12.7)	0.40
Myocardial infarction	0 (0.0)	3 (2.4)	0.36
Respiratory failure	4 (4.8)	9 (7.1)	0.49
Endoleak at pre-discharge CTA			
Type I	27 (32.5)	14 (11.1)	<0.001
- Type Ia	14 (16.9)	8 (6.3)	0.02
- Type Ib	10 (12.0)	5 (3.9)	0.03
- Type Ic	3 (3.6)	1 (0.8)	0.06
Type II	4 (4.8)	6 (4.8)	0.98
Type III	4 (4.8)	9 (7.1)	0.49

## Data Availability

Data are available upon reasonable request to the corresponding author.
